# Improving Production of Treated and Untreated Verbs in Aphasia: A Meta-Analysis

**DOI:** 10.3389/fnhum.2016.00468

**Published:** 2016-09-21

**Authors:** Vânia de Aguiar, Roelien Bastiaanse, Gabriele Miceli

**Affiliations:** ^1^Department of Clinical Speech and Language Studies, Trinity College DublinDublin, Ireland; ^2^Center for Language and Cognition Groningen, University of GroningenGroningen, Netherlands; ^3^Center for Mind/Brain Sciences, University of TrentoRovereto, Italy

**Keywords:** aphasia rehabilitation, verb retrieval, treated and untreated verbs, generalization, predictors of aphasia recovery, neuroplasticity, machine learning, random forests

## Abstract

**Background:** Demographic and clinical predictors of aphasia recovery have been identified in the literature. However, little attention has been devoted to identifying and distinguishing predictors of improvement for different outcomes, e.g., production of treated vs. untreated materials. These outcomes may rely on different mechanisms, and therefore be predicted by different variables. Furthermore, treatment features are not typically accounted for when studying predictors of aphasia recovery. This is partly due to the small numbers of cases reported in studies, but also to limitations of data analysis techniques usually employed.

**Method:** We reviewed the literature on predictors of aphasia recovery, and conducted a meta-analysis of single-case studies designed to assess the efficacy of treatments for verb production. The contribution of demographic, clinical, and treatment-related variables was assessed by means of Random Forests (a machine-learning technique used in classification and regression). Two outcomes were investigated: production of treated (for 142 patients) and untreated verbs (for 166 patients).

**Results:** Improved production of treated verbs was predicted by a three-way interaction of pre-treatment scores on tests for verb comprehension and word repetition, and the frequency of treatment sessions. Improvement in production of untreated verbs was predicted by an interaction including the use of morphological cues, presence of grammatical impairment, pre-treatment scores on a test for noun comprehension, and frequency of treatment sessions.

**Conclusion:** Improvement in the production of treated verbs occurs frequently. It may depend on restoring access to and/or knowledge of lexeme representations, and requires relative sparing of semantic knowledge (as measured by verb comprehension) and phonological output abilities (including working memory, as measured by word repetition). Improvement in the production of untreated verbs has not been reported very often. It may depend on the nature of impaired language representations, and the type of knowledge engaged by treatment: it is more likely to occur where abstract features (semantic and/or grammatical) are damaged and treated.

## Introduction

Aphasia recovery proceeds at a relatively fast pace in the first days after stroke, resolving in 38% of patients. Nonetheless, 43% of patients still present with aphasia 18 months post onset (Laska et al., 2001). Efforts have been made to identify factors that determine the course, the pattern(s) and the potential for language improvement. Though behavioral treatment can substantially change the course of recovery (Pickersgill and Lincoln, 1983), few studies addressed the role played by the deficit targeted by therapy, and the method and content of behavioral treatment. In this meta-analysis we study treatment-related changes in verb retrieval, and identify potential predictors of improvement. We focus on two specific outcomes: improved production of treated and untreated verbs. By including only treatments that required overt verb production, we are able to discuss the role of several potential predictors in relation to the cognitive mechanisms that may be at play during language recovery, for treated and untreated verbs.

### Predictors of aphasia recovery

Several studies identified demographic, clinical and treatment-related variables that may have a predictive value on long-term aphasia severity or functional communication disability. Evidence for the predictive value of these factors will be examined in the following paragraphs. Research on *demographic predictors* indicates better language recovery in younger individuals (Laska et al., 2001; Plowman et al., 2012), in males, and in individuals with high levels of education, socio-economic status, and intelligence (Plowman et al., 2012).

*Clinical predictors* may extend to the pre-stroke clinical history. This way, higher pre-stroke ability to perform everyday activities and duties correlates to better recovery (Maas et al., 2012). Improvement may also be influenced by initial stroke severity (Pedersen et al., 2004; Godecke et al., 2013), lesion site (Plowman et al., 2012), and size (Kertesz et al., 1979; Maas et al., 2012; Plowman et al., 2012). Recently, it was suggested that lesion size does affect recovery, but only to the extent in which larger lesions are more likely to encompass critical anatomical areas (Price et al., 2010).

Lesion size is thought to be inversely related to the role of intact peri-lesional and contra-lesional brain areas in recovery. In neuroimaging studies, increased activation in post- vs. pre-treatment comparisons has been observed in left frontal and posterior temporo-parietal areas, in association with improved language performance (Fridriksson et al., 2012). In addition, while some right-hemisphere areas may have a disruptive influence on left hemisphere functions, others may contribute to better language processing (Turkeltaub et al., 2012). For example, a larger volume of the long segment of the right arcuate fasciculus predicts the amelioration of the aphasia quotient (Forkel et al., 2014).

Time post-onset is typically considered a relevant predictor of recovery, based on the observations of spontaneous recovery in the first months after stroke (e.g., Laska et al., 2001). However, in a single-case meta-analysis, Moss and Nicholas (2006) found no correlation between time post-onset and degree of improvement in individuals who had began treatment 1 year after stroke.

Cognitive variables relate to the patient's cognitive profile after stroke. Across studies, initial aphasia severity was consistently identified as a predictor of language improvement (Pedersen et al., 1995, 2004; Plowman et al., 2012; Godecke et al., 2013). More specifically, studies report on the predictive roles of functional communication abilities at onset (Ramsing et al., 1991; Laska et al., 2001) and of the initial severity of phonological impairment (as measured by tasks such as repetition, reading aloud, same/different judgments with auditorily-presented word pairs, and matching the first phoneme of a spoken word with a grapheme; El Hachioui et al., 2013). Severity also determines the recovery path, as stationary performance can be reached as early as 2 weeks post-onset by individuals with mild aphasia, at 6 weeks by those with moderate aphasia, and at 10 weeks by those with severe aphasia (Pedersen et al., 1995). Pickersgill and Lincoln (1983) suggested that recovery of language modalities follows a specific pattern, in which comprehension improves before production. Accordingly, different courses of recovery were reported for patients with intact and with impaired comprehension, the former improving in speech production and the latter in comprehension and word repetition (Lomas and Kertesz, 1978). Visuo-motor speed and attention predict return to work (Ramsing et al., 1991).

Few studies have addressed the characteristics of treatment that predict better recovery. However, there is evidence that aphasia rehabilitation is effective both in the acute and in the chronic stages (De Jong-Hagelstein et al., 2011; Brady et al., 2012). In fact, treatment may substantially change the course of recovery. Patients who undergo Speech-Language Therapy improve more rapidly than those who do not. This difference is particularly evident in the first 4 months after stroke (Pickersgill and Lincoln, 1983). This way, features of treatment may be considered *treatment-related predictors* of recovery. One predictor of recovery that has been identified is the intensity of treatment: better outcomes were associated with more intense aphasia therapy and with a higher number of total treatment hours (Bhogal et al., 2003; Godecke et al., 2013).

Jacquemot et al. (2012) carried out a meta-analysis of single-case treatment outcomes to identify the features of treatment relevant for improvement. The meta-analysis reported that only tasks that engaged output phonology contributed significantly to naming improvement. In addition, they showed that treatment is more effective when it addresses the impaired level of language processing. These results highlight the specificity of improvement in relation to the levels of language processing engaged by treatment, and in relation to those levels affected by neurocognitive damage.

Hickin et al. (2002) investigated the predictive value of cognitive factors in recovery while taking into account the tasks used during treatment. They found that the effects of facilitation (the degree of priming obtained from a single exposure to a cue) correlate with effects of treatment (improvement observed with the repeated administration of the same cue). In aphasia, effects of semantic priming (Baum, 1997) and repetition priming on lexical retrieval have been reported (Nickels, 2002). The data by Hickin et al. (2002) indicate that the degree of priming predicts the potential for recovery if the same task is used in treatment, therefore strengthening the argument that it is relevant to examine predictors of aphasia recovery while taking into account the characteristics of treatment.

On a final note regarding predictors of aphasia recovery identified in the literature, it is crucial to consider that the outcome predicted may vary across studies. This way, it is important to clearly define what recovery means in a particular study. The meta-analysis of Dickey and Yoo (2010) illustrates the importance of outcomes. They studied inter-individual differences in response to linguistically motivated aphasia therapy. Treatment protocols of this type promote explicit, meta-linguistic knowledge of language structure, which can generalize to untreated materials, and therefore promote more widespread language improvement (Thompson and Shapiro, 2005). In the study by Dickey and Yoo (2010), auditory comprehension scores predicted improvement for treated sentences, but none of the examined variables predicted generalization to untreated sentences. These results suggest that improvement for treated and untreated materials may rely on different neurofunctional mechanisms (Dickey and Yoo, 2010) and may, therefore, be predicted by different variables.

#### The process of verb production

While a detailed description of the language processing system is beyond the scope of this report (we refer the reader to Patterson and Shewell, 1987; Dell, 1988; Gagnon et al., 1997; Miozzo and Caramazza, 1997; Levelt, 1999; Foygel and Dell, 2000; Rapp and Goldrick, 2000; Bastiaanse and Van Zonneveld, 2004), a schematic summary of the mechanisms involved in language production is important to understand the functional effects of therapy for verb retrieval. Different language models acknowledge the existence of conceptual features (that is, the semantic features that generate meaning and, for a verb, its thematic roles), syntactic features (that is, a set of grammatical features such as grammatical class, noun gender, verb argument structure, subcategorization frame, etc.), and phonological representations (that is, the segmental and supra-segmental properties of the word's phonology). Models differ in whether phonological and syntactic features are thought to be accessed sequentially (e.g., Levelt, 1999; Bastiaanse and Van Zonneveld, 2004) or in parallel (e.g., Patterson and Shewell, 1987; Miozzo and Caramazza, 1997), and in whether the interaction between different levels of representation is considered unidirectional (Patterson and Shewell, 1987; Miozzo and Caramazza, 1997; Levelt, 1999; Bastiaanse and Van Zonneveld, 2004) or bidirectional (Dell, 1988; Rapp and Goldrick, 2000).

**Figure 1:**
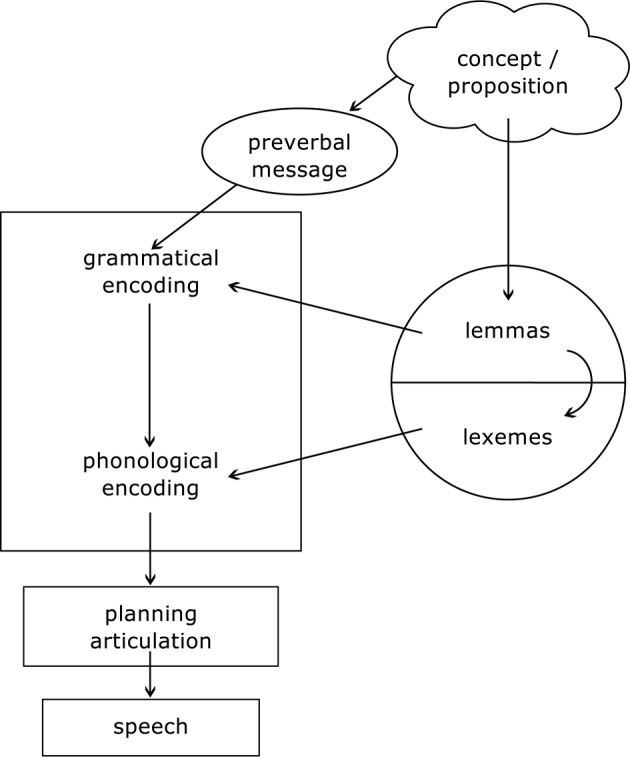
**Schematic representation of a language processing model**. Based on Bastiaanse and Van Zonneveld (2004), and adapted from Levelt (1989). Copyright: Prof. Roelien Bastiaanse, University of Groningen.

As an example, we present the model introduced by Bastiaanse and Van Zonneveld (2004, adapted from Levelt, 1989), illustrated in Figure 1. According to this model, when a concept is triggered, it will activate a *lemma*. In this model, the lemma level includes both lexical-semantic (meaning) and syntactic information. In other sequential models it is assumed that a concept triggers retrieval of semantic representations, and syntactic features are activated subsequently. In these competing models, activation of syntactic features occurs either before the retrieval of lexemes (Levelt, 1999) or at the lexeme level (Miozzo and Caramazza, 1997).

According to Bastiaanse and Van Zonneveld (2004), the *grammatical encoder* receives input from two sources (preverbal message and lemma level) and uses this information to form a sentence frame. The idea that a speaker wants to express (which may be the name of an object or action, but also a complete proposition) is formulated in a preverbal message. The grammatical encoder uses the verb-argument structure represented in the verb lemma to generate a sentence frame that suits the grammatical properties of the verb and the intention of the speaker (the concept / proposition). Therefore, it also specifies which grammatical information should be filled in the sentence frame, for example, 3rd person singular/plural; past/present tense etc. The lemma activates the *lexeme* (that is, lexical-phonological representation or phonological word form), which is inserted in the sentence frame constructed by the grammatical encoder. This is the process of phonological encoding: phonemes are inserted and phonological rules are applied to plan and execute the articulation process. Verb production deficits may reflect impairment at each of the levels and processes described in this model.

#### Recovery of verb production

The syntactic information associated with verbs is necessary for the production of grammatically well-formed sentences (Saffran et al., 1980). Accordingly, verb production scores are better predictors of communication in daily living than noun production scores, when both word classes are produced in sentence context (Rofes et al., 2015). Though relevant for everyday communication, and selectively impaired in a considerable number of patients (e.g., Miceli et al., 1984; Luzzatti et al., 2002; Shapiro and Caramazza, 2003; Benetello et al., 2016), verb production has been less often targeted in treatment studies than noun production.

A recent review shows that at the single-word level, verb retrieval disorders can be treated using the same Speech-Language Therapy techniques used for the treatment of noun retrieval (Webster and Whitworth, 2012), but suggests that verb recovery is more difficult to achieve. With verbs and nouns differing at the levels of semantic and grammatical detail that are entailed in their representations (see Webster et al., 2004; Conroy et al., 2006; Maguire et al., 2015), it is possible that the difference in treatment efficacy means that verb recovery and noun recovery rely on different mechanisms. A full investigation of factors that determine verb recovery is yet to be carried out. In examining these factors, the nature of the outcome (e.g., improvement in treated vs. untreated verbs) must be considered.

As suggested by Dickey and Yoo (2010) improvement in production of treated and untreated items relies on different mechanisms. Generalization^1^ is seldom reported in the aphasia rehabilitation literature, though it was observed after treatment of argument structure (Thompson et al., 2013), and of tense production in sentences (Links et al., 2010; de Aguiar et al., 2015). It has been proposed that generalization is constrained by the underlying cognitive impairment (it would be more likely in the event of semantic impairment, than in the event of lexeme-level damage; Miceli et al., 1996) and/or by characteristics of the therapy task (more likely when abstract semantic or syntactic features are treated; e.g., Boyle and Coelho, 1995; Thompson and Shapiro, 2005). In addition, it has been proposed that generalization is also influenced by an interaction of linguistic and extra-linguistic computations: with practice, the treatment task becomes easier and the cognitive load of task-specific computations is reduced. Consequently, more processing resources can be allocated to lexical retrieval when treated and untreated items are presented in the same task (de Aguiar et al., 2015).

Although some accounts for the cognitive mechanisms of improvement in aphasia have been proposed (e.g., Boyle and Coelho, 1995; Miceli et al., 1996; Thompson and Shapiro, 2005), a systematic evaluation of demographic, clinical, anatomical and treatment-related variables that may influence outcome is lacking. We report on a meta-analysis of single-case studies and single-case series in which the treatment task required overt verb production. We examine the predictive value of demographic, clinical, and treatment-related factors in determining treatment outcome, weighing the relative contribution of each variable while taking into account all the others. The potential contribution of these factors to treatment outcome is assessed for improvement in production of treated and untreated verbs. We discuss the potential cognitive mechanisms of change in response to treatment that each variable may reflect.

## Predictors of aphasia recovery

### Method

#### Data extraction from the literature

We conducted a web search using the main search engines (Pubmed, Web of Science, and Google Scholar). We searched for articles including the key words Aphasia rehabilitation/treatment AND verbs OR Aphasia rehabilitation/treatment AND actions OR Aphasia rehabilitation/treatment AND sentences. We excluded literature reviews, neuromodulation studies, and articles in which (1) the aphasia rehabilitation technique did not entail overt verb production, (2) pre- and post-treatment performance was only measured in terms of morphosyntactic accuracy (rather than accuracy in lexical retrieval of verbs), and (3) no statistical analysis was reported on the outcomes of treatment for each individual. We considered only post-stroke aphasia, and excluded cases with other neurological conditions (e.g., head traumas, tumors, primary progressive aphasias, etc.). The final database included 166 individual treatment outcomes, obtained from 30 articles^2^.

From each study, we extracted the outcome of each treatment for each patient. The analyzed outcomes include improvement in retrieval of treated verbs (presence/absence of significant improvement), improvement in retrieval of untreated verbs (presence/absence of significant improvement). Significant improvement was considered present based on criteria employed by the authors of each study, but only when appropriate statistical procedures were used. While the tasks used to measure improvement varied across studies (e.g., sentence construction in Weinrich et al., 1997 and action naming in Conroy et al., 2009a), responses had to be scored for accuracy in lexical retrieval of the verb to meet our inclusion criteria.

For each patient, we extracted three types of predictors: demographic, clinical, and treatment-related. Demographic variables included Age, Gender, and Education. Clinical variables were Months Post-Onset, pre-treatment assessment scores (Noun Production, Verb Production, Noun Comprehension, Verb Comprehension, Word Repetition, Nonword Repetition), and variables relating to pre-treatment diagnosis (Fluency, Semantic Impairment, Lexeme Impairment, Sublexical Processing Impairment, and Grammatical Impairment).

The focus on pre-treatment assessment scores, rather than a compound measure of aphasia severity (e.g., El Hachioui et al., 2013) was adopted to provide a more specific account regarding the aspects of impairment (and its severity) that may predict recovery. For example, the severity of the comprehension impairment may show interactions with other variables that are not related to the severity of the naming impairment. These scores were obtained from a variety of standardized language batteries (e.g., Object and Action Naming Battery: Druks and Masterson, 2000; Verb And Sentence Test: Bastiaanse et al., 2002), and from experimental tasks designed for the specific purposes of each study (e.g., Weinrich et al., 1997; Maul et al., 2014). Due to the lack of normative data in several *ad-hoc* tests, score normalization was not possible. Instead, percent accuracy in each task was calculated.

The decision to consider several variables related to pre-treatment diagnosis (e.g., presence of semantic damage) was motivated by the observation that the presence/absence of comprehension impairment determines the type of recovery (Lomas and Kertesz, 1978). Our aim was to explore whether descriptions of impairment more specific than overall comprehension scores would highlight specific aspects of comprehension (and production) that determine the outcome of therapy. Data for each of these diagnostic variables were inserted in our data as described in the article, when available. When diagnostic information was not explicitly reported, but available data allowed reasonable hypotheses about the potential loci of language impairment, such information was produced by the authors using the methodology outlined in Whitworth et al. (2014). In this approach, hypotheses about the locus of impairment within the language processing system are tested by observing the performance on tasks that share levels of processing, the nature of errors, and the effect of psycholinguistic variables on performance. This approach assumes functional modularity, anatomical modularity, and universality of the language processing system, as well as subtractivity of processing components in the event of a lesion.

As mentioned in the introduction, treatment-related variables are not typically taken into account in studies focused on predictors of recovery. However, the methodology here employed allows identifying interactions between predictors of different nature. Therefore, we included treatment-related variables that reflected the treatment tasks administered in the different studies that contributed to our dataset. These included the Level of Output required of the patient (single words, sentences, or both), the Level of Input provided by the therapist (cues consisting of single words, sentences, or both), Cue Direction (increasing/decreasing cues), Finite Verbs (administration of a treatment task targeting/not the production of verbs in more than one finite form), Semantic Cues (therapist providing/not semantically loaded sentences to facilitate word retrieval), Phonemic Cues (therapist providing/not the initial phonemes of the target word), Repetition Cues (therapist providing/not the target word for repetition), Written Cues (therapist providing/not the target word in written form), Morphological Cues (therapy technique including/not specific training of tense morphology, with verbs produced in a variety of tenses), knowledge of verb Argument Structure (target response requiring/not knowledge of the predicate argument structure of the verb), Gestural Cues (therapist presenting/not a gesture facilitative of target retrieval).

Other treatment-related features included in the analyses addressed dimensions of dose, frequency and intensity, similarly to those examined in previous research (e.g., Bhogal et al., 2003). These were: Number of Treatment Sessions, Number of Treatment Days (the period across which treatment was delivered), Total Number of Treatment Hours (collapsed across all treatment sessions), Session Duration (in minutes), Session Frequency (number of sessions per week), and Treatment Intensity (number of hours per week). Furthermore, when predicting improvement for untreated verbs, improvement of treated verbs was included as one of the potential predictors.

#### Statistical analyses

Results were analyzed by means of the Random Forests. Random forests is a machine-learning algorithm used for classification and regression. This methodology is particularly suitable for the analysis of data with many variables of different types (both continuous and categorical) and relatively few cases (Breiman, 2002; Liaw and Wiener, 2002). This method was selected because other advanced statistical treatment methodologies, such as logistic mixed regression models, could not compute models that account for complex interactions with many variables and few cases with the same reliability (for a demonstration of the superiority of random forests in modeling linguistic data, see Tagliamonte and Baayen, 2012). An additional reason for using Random forests is that it allows to extract variable importance. This dimension reflects the average reduction of a model's accuracy when a given variable is left out (Breiman, 2002). This technique is widely used in genetic research (Díaz-Uriarte and De Andres, 2006), but to the best of our knowledge this is the first attempt to model predictors of aphasia recovery using Random Forests.

**Figure 2:**
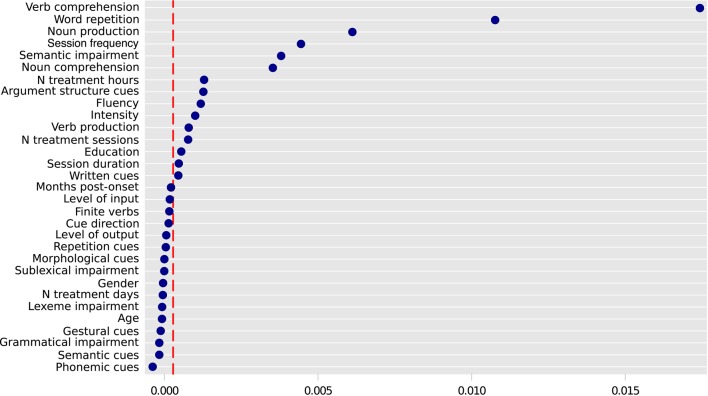
**Variable importance for improvement of lexical retrieval for treated verbs**. Predictors to the right of the dashed vertical line are potentially relevant and informative. Variable importance varies randomly around zero, and therefore predictors to the left of the red line are not informative. The absolute values of variable importance cannot be compared across different Random Forest models (e.g., the ones represented in Figures 2, 4). Only the descriptive ranking of the most important variables is relevant here (Strobl et al., 2009b). Variable importance is presented in the x axis.

Data preparation and statistical analysis followed these steps, for each outcome variable:

1. Missing values were imputed (that is, estimated) using Random Forests with the function rfImpute (Breiman, 2002; Liaw and Wiener, 2002). For factors, missing values are initially replaced by the most frequent level (breaking ties at random), and then adjusted based on a proximity matrix (that is, a measure of similarities across cases, that considers information available from other variables). Estimates were based on 100 iterations of growing 2000 trees. For additional quality check, this procedure was repeated 20 times, hence creating 20 different databases. The quality of estimated data was ensured by examining the consistency of the results obtained with different imputations. This procedure has been reported to produce accurate predictions in samples with a missingness of up to 56% (Shah et al., 2014). Therefore, we excluded variables with proportions of missingness above this value.
2. For each database, a random forest was computed by using the cforest function (Hothorn et al., 2006a). We then extracted the importance of each potential predictor in determining outcome (varImp function: Strobl et al., 2009a). A conditional permutation importance was used to maintain the accuracy of predictions in the presence of correlations between variables (Strobl et al., 2009a).
3. The importance attributed to each potential predictor was averaged across the 20 data imputations, and the *z*-value of the importance of each variable was calculated in each of the 20 data sets. The dataset and the random forest that produced variable importance measurements closest to the mean of the 20 imputations were selected for further analyses.
4. Following the procedures in Tagliamonte and Baayen (2012), the accuracy (index of concordance *C*) of the selected random forest was calculated by using treeresponse (Hothorn et al., 2015). A *C*-value above 0.80 indicates good classification performance (Chatterjee and Hadi, 2006).
5. Finally, the ctree function (Hothorn et al., 2006b) was used to construct conditional inference trees that illustrate how different predictors interact. Variables to include in the model were selected using a backwards elimination procedure, following the same principles adopted in gene selection studies (Díaz-Uriarte and De Andres, 2006). Initially, all variables were included in the conditional inference tree. Iteratively, the least important variable was removed from the model. A variable was selected to remain in the model when its removal resulted in a decrease in model accuracy, measured with tree response. The best conditional inference tree is reported in Figures 2, **4**. Example R code is available in the Appendix.

## Results

### Improvement of lexical retrieval for treated verbs

We extracted from the literature 142 cases in which treatment outcomes were reported for treated verbs. Significant improvement in verb retrieval was reported for 108 cases (76.1%), whereas 34 cases (23.9%) showed no treatment effect. The variable Nonword Repetition was not included in the Random Forest for treated verbs, due to a large proportion of missing data. For treated verbs the percentage of missing data was of 37% for the Number of treatment hours and Session duration, 35% for Treatment Intensity, 26% for Word repetition, 24% for Noun Comprehension, 18% for Verb Comprehension, 12% for Education, and 10% for Noun production. For untreated verbs, the percentage of missing data was of 45% for Nonword repetition, 41% for Word repetition, 40% for Noun comprehension, 31% for Total Number of Treatment Hours, 31% for Session Duration, 30% for Intensity, 28% for Education, 21% for Improvement for treated verbs, 20% for Semantic Impairment and for Lexeme Impairment, and 8% for Noun Production. All other variables had missingness rates below 5%, both for treated and untreated verbs. After selecting the most representative imputed dataset using procedures (1) to (3) (see Method section), we obtained a random forest with an index of concordance *C* = 0.94, and an Out-Of-Bag error (that is, classification error rate) of 0.18. The variable importance is represented in Figure 2.

**Figure 3:**
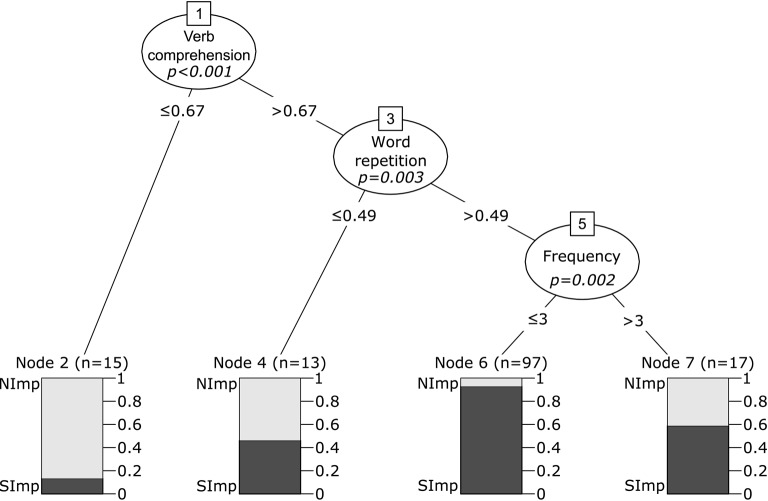
**Conditional inference tree for treatment outcome for treated verbs**. Nodes 1, 3, and 5 represent significant variables, with *p*-values presented within the node. Below each of these nodes, the values represent the points at which the node splits, therefore separating patients in groups with different outcome predictions. Each of these groups is represented by a box, and the colors in the box represent the proportion of patients within each group that showed significant improvement after treatment (SImp, in dark gray) and the proportion of patients that did not (NImp, in light gray).

The nodes of the tree in Figure 3 split automatically, based on differences in the probability of improvement observed for the different levels of a factor (e.g., the “Frequency” node, numbered 5 in Figure 3). For continuous variables, the values that determine the split of the node are estimated on the basis of two-sample standardized statistics (Hothorn et al., 2006b). The bars at the bottom of the tree represent the proportion of patients who improve and who do not improve, at each node of the tree. The split in verb comprehension around 67% accuracy indicates that patients with very poor verb comprehension (< 67% correct on a comprehension test) were less likely to show item-specific improvement than those with verb comprehension accuracy above 67% (note the low proportion of patients who showed item-specific improvement, in the left-most branch of the tree). As for patients with comprehension above 67% accuracy, the subgroup with very poor repetition (< 49% in word repetition test) was less likely to improve than the one with repetition accuracy above 49%. Among the latter, the subgroup that received fewer than three therapy sessions per week was more likely to improve than the subgroup receiving more than three sessions per week.

### Improvement of lexical retrieval for untreated verbs

**Figure 4:**
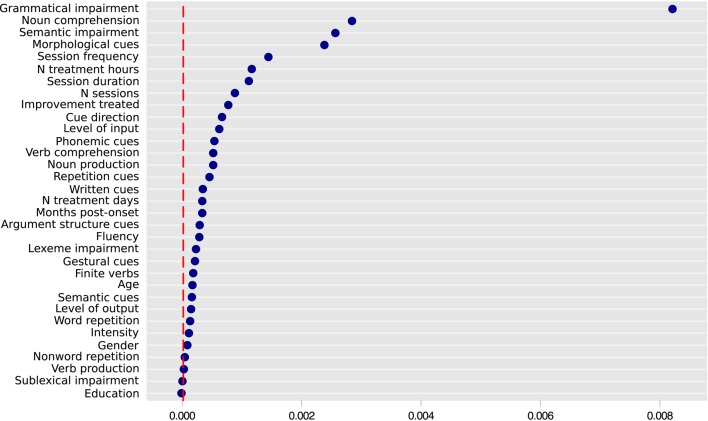
**Variable importance for improvement of retrieval for untreated verbs**. Predictors to the right of the dashed vertical line are potentially relevant and informative. Variable importance varies randomly around zero, and therefore predictors to the left of the red line are not informative. The absolute values of variable importance cannot be compared across different Random Forest models (e.g., the ones represented in Figures 2, 4). Only the descriptive ranking of the most important variables is relevant here (Strobl et al., 2009b). Variable importance is presented in the x axis.

**Figure 5:**
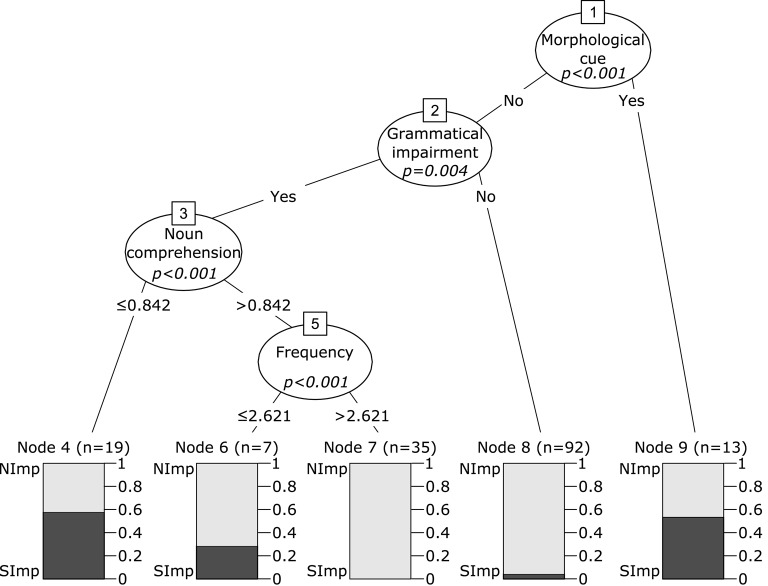
**Conditional inference tree for treatment outcome for untreated verbs**. Nodes 1, 2, 3, and 5 represent significant variables, with *p*-values presented within the node. Below each of these nodes, the values represent the points at which the node splits, therefore separating patients in groups with different outcome predictions. Each of these groups is represented by a box, and the colors in the box represent the proportion of patients within each group that showed significant improvement after treatment (SImp, in dark gray) and the proportion of patients that did not (NImp, in light gray).

The binary outcome for untreated verbs was reported for 166 patients. Significant improvement was observed in 24 cases (14.5%), whereas 142 (85.5%) individuals did not show improvement in the production of untreated verbs. The most representative imputed dataset produced a random forest with an index of concordance *C* = 0.96, and an Out-Of-Bag error rate of 0.14. The variable importance for predicting improvement in untreated verbs is represented in Figure 4. The best conditional inference tree was produced with the variables Grammatical Impairment (*p* = 0.004), Noun Comprehension (*p* < 0.001), Morphological Cue (*p* < 0.001), and Frequency (*p* < 0.001), reaching *C* = 0.88 (Figure 5). No other variables met the established criteria.

The two branches of the Morphological Cue node show that patients whose treatment protocol included morphological cues (consisting in all cases of therapy for tense production) were more likely to show improvement for untreated verbs. Among those who did not receive morphological cues during treatment, greater chances of improvement were observed in patients with grammatical impairment. In this subgroup, patients with poorer noun comprehension were more likely to improve than those with higher scores (< 84.2% accuracy in a noun comprehension test). Finally, patients with relatively spared comprehension were more likely to improve when they received fewer than 2.6 therapy sessions per week. A closer examination of the data shows that all patients who received morphological cues had been diagnosed with grammatical impairment. We compared improvement in patients with grammatical impairment who received (node 9 of the tree in Figure 5; *n* = 13) and who did not receive morphological cues (node 3; *n* = 61). Patients who did not receive morphological cues were less likely to improve [*X*^2^(1, *N* = 74) = 4.22, *p* = 0.039].

## Discussion

Our meta-analysis of the literature on verb rehabilitation highlights differences between the frequency of occurrence and the predictors of improvement for treated and untreated verbs. Improvement of lexical retrieval for treated verbs was observed in 76.1% of the cases. It occurred more often in patients with higher verb comprehension scores. Among them, those with word repetition accuracy above 49% improved more often than those with poorer repetition. In patients with relatively high verb comprehension and word repetition scores, improvement was more likely when they received fewer than 3 therapy sessions per week. Improved production of untreated verbs was uncommon (14.5% of the sample). It was observed more frequently in patients whose treatment included morphological cues. A specific pattern was observed in patients who did not receive morphological cues: improvement was more frequent in individuals with grammatical impairment and poor noun comprehension (< 85%). Patients with grammatical impairment and relatively high noun comprehension were more likely to improve if they received fewer than 2.6 treatment sessions per week.

In the next sections we discuss the nature of recovery processes that may explain the role of these predictors. We start by discussing variables that are specifically relevant for either item-specific improvement or for generalization, and then discuss Frequency, which is common to both types of outcome.

### Improvement of lexical retrieval of treated verbs

Patients who perform well on standardized language tests show a higher potential for improvement. This has been reported frequently in studies showing that severity plays a role in predicting recovery (e.g., Pedersen et al., 2004; El Hachioui et al., 2013; Godecke et al., 2013). Dickey and Yoo (2010) examined more specific predictors (aphasia severity, but also general auditory comprehension and comprehension of complex sentences) and a more specific outcome (improvement in sentence production). They report that auditory comprehension predicts improvement in the production of treated sentences. In their study, aphasia severity as measured by the aphasia quotient did not predict improvement in the production of treated or untreated sentences. They interpreted this finding as indication that a measure of severity that is too broad may not capture the mechanisms of change induced by a treatment targeting specific linguistic processes, and therefore may fail to correctly predict treatment outcome. In our study, the measures that capture the severity dimension were very specific (individual assessment tasks). Pre-treatment verb production did not surface as a crucial predictor. In the following paragraphs, we will argue that the observed predictive value of verb comprehension and word repetition should not be reduced to an effect of severity, but may rather reflect aspects of cognition that participate in recovery for treated items.

Both in response to treatment (Pickersgill and Lincoln, 1983), and in spontaneous recovery (Lomas and Kertesz, 1978), the improvement of comprehension has been reported to precede that of production. Lomas and Kertesz (1978) propose that due to the broader representation of receptive language in relation to production, severe comprehension impairment may be associated with larger lesions and greater overall stroke severity. However, they also report that severity of the comprehension impairment determines not only the amount, but also the type of improvement: patients with poor comprehension improve mainly in repetition and comprehension whereas improvement in language production occurs mostly in those with good comprehension. This suggests that different mechanisms of change may be at work in patients with different levels of comprehension.

In the extracted data, verb comprehension was typically measured by testing the ability to match an auditorily-presented word to a picture, presented in an array that includes the target and one or more distractors (e.g., unrelated or semantic foils such as “rowing” for the target “sailing”; Druks and Masterson, 2000; Bastiaanse et al., 2003). Accurate performance on this task requires a complex set of processes. The input string of sounds/letters must be analyzed and recognized as a word in the appropriate input lexicon, and must activate the corresponding meaning (Patterson and Shewell, 1987). At the same time, the action pictures must activate abstract visuoperceptual representations and, subsequently, their corresponding meaning. Typically, pictures are selected so as to share many semantic features. The correct response is then contingent upon the ability to select the picture whose meaning fully matches that of the stimulus word. Poor performance on this task can therefore reflect deficits that arise at each of these levels of processing.

The predictive value of comprehension scores may result from both a generic and a specific influence on verb retrieval. At a very general level, poor comprehension may significantly disrupt the therapeutic process by hampering participation in treatment tasks and successful implementation of compensatory strategies. The predictive effect of comprehension can be more specific, however. Semantic knowledge is involved both in word-picture matching and in action naming. Therefore, a severe impairment of semantic knowledge will inevitably yield deficits of both verb comprehension and production in the same individual. In agreement with this possibility, prior research showed that lexical processing can be facilitated by semantic priming in individuals with aphasia and that the priming effect is reduced or absent in patients with poor comprehension (Baum, 1997). According to models that assume that the semantic features associated with a concept are activated before the corresponding lexeme (e.g., Patterson and Shewell, 1987; Miozzo and Caramazza, 1997; Levelt, 1999; Bastiaanse and Van Zonneveld, 2004), the probability that a lexeme is activated above threshold is constrained by the level of activation of semantic representations. If semantic information is available^3^, activation can feedforward to the lexeme level, leading to successful retrieval of lexical forms. Conversely, in individuals with impaired semantic representations, target lexemes will be less likely to be activated above threshold levels. This means that the opportunity to practice the retrieval of lexemes is reduced when semantic information is substantially unavailable.

Patients with relatively preserved verb comprehension (above 67% accuracy) and with word repetition scores >49% had an increased chance of improvement. Also in this case, the relationships between verb retrieval, verb comprehension, and word repetition can be complex. An obvious possibility is to attribute the predictive value of repetition to the fact that tasks of this type rely on Short-Term Memory (STM; Baldo et al., 2008). In this framework, it should be stressed that influential models of memory (e.g., Atkinson and Shiffrin, 1968), posit a crucial role for STM in long-term learning. In agreement with this view, Papagno et al. (1991) showed that healthy individuals use the phonological loop (in particular, subvocal rehearsal) when learning a foreign language vocabulary. In addition, damage to phonological STM (disrupting phonological recoding and phonological rehearsal) impedes learning of new vocabulary (in a foreign language), regardless of long-term learning abilities (Baddeley et al., 1988). Considering the relation between word repetition and phonological short-term memory, and the support that short-term memory provides to vocabulary learning, our results suggest that short-term memory processes/abilities indexed by word repetition may facilitate restoration of (or access to) lexemes. The exact nature of these processes cannot be established based on word repetition alone. Future research should address the relation between STM and aphasia recovery using more direct measures of working memory.

Interestingly, STM (as measured by repetition scores) affects performance only in a subset of patients with relatively good verb comprehension (see Figure 3). If poor comprehension disrupts the therapeutic process (as discussed in a previous paragraph), it is possible that the STM-mediated mechanisms of improvement are only effective if the patient's level of comprehension allows therapy to proceed efficiently. That is, if the patient does not understand the therapy task and the task cannot be implemented, practice cannot proceed and good STM may not produce benefits. In addition, one should consider that regardless of STM skills, picture-elicited verb retrieval requires access to lexemes. Perhaps relatively spared STM can facilitate recovery only in the presence of good comprehension because, in this case, intact access to the output lexicon can be strengthened to facilitate lexical selection (Baum, 1997). Supporting evidence comes from the repetition priming literature: priming effects are stronger (that is, they last longer) for words than for nonwords (Kirsner and Smith, 1974; Scarborough et al., 1977; Dannenbring and Briand, 1982), and with nonwords they occur only on items included in a previous study phase (Sereno, 1991). Finally, Lomas and Kertesz (1978) reported that aphasic patients with poor comprehension improve in repetition, while their production skills remain unchanged after therapy. This reinforces the hypothesis that repetition and its correlated cognitive processes only serve as a resource to facilitate recovery of lexical retrieval when access to the lexeme is at least partly spared. If it is not, rehabilitation of comprehension should be a priority.

The predictive value of repetition scores may also accrue from damage to mechanisms other than short-term memory, such as segmental disorders (the inability to retrieve the target phonemes, or to produce them in the correct order), or apraxia of speech (the inability to convert an abstract phonological representation into a correct speech plan). Protocols aiming at the recovery of verb retrieval deficits and focusing on overt language production (such as those selected for the present meta-analysis) have a greater chance of success when post-lexical damage is absent or mild, as in this case restoring activation of the lexemes targeted by treatment suffices to allow correct responses to occur. By contrast, damage to segmental (phonological) information or apraxia of speech will interfere with speech output. If sufficiently severe, post-lexical impairments may render verb retrieval remediation protocols ineffective by disrupting responses at the segmental level. This would yield both poor verb production and low repetition scores.

### Improvement of lexical retrieval of untreated verbs

The factors that influence improvement in lexical retrieval of untreated verbs differ from those associated with improvement in retrieval of treated verbs. Prior studies had already suggested that item practice and task practice may rely on different neurocognitive mechanisms (Basso et al., 2013). The factors that significantly predict improvement in production of untreated verbs are diverse. Two of them relate to treatment characteristics (morphological cues, frequency) and two to features of the subject's language impairment (noun comprehension, frequency). In the next paragraphs, we discuss treatment-related cognitive changes that may account for the predictive value of these variables.

Generalization in verb retrieval occurs infrequently (Webster and Whitworth, 2012), even following treatment techniques shown to result in generalization to untreated nouns, such as Semantic Feature Analysis (Wambaugh and Ferguson, 2007; Wambaugh et al., 2014). It is more likely when treatment addresses abstract properties or rules (e.g., argument structure or inflectional paradigm) that apply to more than one word or sentence. In the intact language system, different verbs share information about the syntactic structures in which they occur (Pickering and Branigan, 1998), and this facilitates production of shared constructions (structural priming: e.g., Bock, 1986). In aphasia, generalization is reported in the lexical retrieval of verbs after treatment of argument structure (Thompson et al., 2013), and of tense production in sentences (Links et al., 2010; de Aguiar et al., 2015). Our findings are in line with these studies, as patients with grammatical impairment who did not receive morphological training were less likely to improve in the production of untreated verbs than those who did. Training of these abstract properties, and in particular morphological training, may facilitate verb retrieval by alleviating the cognitive load associated with encoding grammatical information, thus allowing more resources to be allocated to verb retrieval. Data in line with this account were reported by Bastiaanse and Jonkers (1998) for agrammatic and Bastiaanse (2011) for fluent aphasic speakers. In spontaneous speech of both aphasic subgroups there is a relation between morphosyntactic complexity and verb retrieval.

Miceli et al. (1996) propose that generalization may or may not occur depending on the nature of the cognitive processes and representations that are impaired. For instance, lexemes are unique labels in an individual's mental lexicon, each specifying the phonological form associated with a concept (e.g., Roelofs et al., 1998). If a patient has a deficit specific to lexical representations, treating a word is unlikely to improve retrieval of a different word, and item-specific improvement without (or with minimal) generalization to untreated items is expected. In agreement with the prediction, Miceli et al. (1996) found no generalization (even to semantically-related words) in two patients with anomia due to lexical damage. Similar results were obtained by Fillingham et al. (2006), Hickin et al. (2002), and Parkin et al. (1998).

A different outcome can be expected when information shared by many items is unavailable. As discussed above, restoration of grammatical information using a small set of treated items can make the same knowledge available for untreated verbs (Links et al., 2010; Thompson et al., 2013; de Aguiar et al., 2015). However, patients may only benefit from this transfer of knowledge if they are lacking it before treatment. As reflected by our sample, morphosyntactic cues are typically presented to patients with “agrammatic” aphasia. These patients are more likely to show improvement for untreated verbs following verb therapies than patients without grammatical impairment. This is true even if patients do not receive treatment specific to grammatical processing: in the subgroup of patients who did not receive morphological cueing, patients with grammatical impairment were still more likely to generalize than those without grammatical impairment. This finding supports the claim that improvement for untreated verbs depends (at least partially) on the nature of the affected language processes (Miceli et al., 1996), as change is more likely to be measurable if the function under scrutiny is impaired.

We should, however, keep in mind that even when morphological cues were not presented, treatment may have engaged other types of grammatical knowledge. In fact, theories of speech processing share the claim that syntactic features (e.g., grammatical class, agreement, and case assignment, etc.) are stored independently from the corresponding phonological form (Miozzo and Caramazza, 1997; Roelofs et al., 1998; Bastiaanse and Van Zonneveld, 2004), and are retrieved even when verbs are produced as isolated words. While some studies suggest that sentence level treatment may be more conducive to generalization (Links et al., 2010; Thompson et al., 2013; de Aguiar et al., 2015), in the current study, the level of input (receiving cues as single words, sentences, or both) and the level of output (producing single words, sentences, or both) did not feature in the interactions of variables predicting either outcome. Rather, syntactic features may be accessed implicitly even when treatment does not overtly require it and even if it is provided at the single word level. Patients with grammatical impairment may be more prone to generalization, due to the impairment of shared (hence, generalizable) syntactic features, which are implicitly engaged in treatment and can be at least partially restored.

Just like verb comprehension, poor noun comprehension may reflect damage at different levels of processing. Comprehension predicted improvement in opposite directions for treated and untreated verbs: while patients with low verb comprehension scores improved less often on treated verbs, patients with low scores on tests for noun comprehension (and grammatical impairment) were more likely to improve on untreated verbs. In short, the data cannot be accounted for by widespread comprehension impairment. As argued earlier for verb comprehension tests, noun comprehension tests also extensively engage semantic representations. However, why damage to meaning representations was reflected by comprehension scores for nouns rather than verbs remains puzzling. We provide two tentative explanations for this. Firstly, this could happen because noun representations, in comparison to verb representations, have particularly high semantic detail (Conroy et al., 2006; Maguire et al., 2015). Therefore, performance in tests that use nouns may be more dependent on semantic abilities, and noun tests may then be particularly sensitive in detecting impairment in semantic knowledge. A second explanation is of methodological nature. Importance ratings show both verb and noun comprehension to be informative in predicting improvement on untreated verbs. However, when considered simultaneously, one surfaces as more important, possibly because both variables account for similar dimensions but one (in this case, noun comprehension) is more sensitive (Ishwaran et al., 2008).

Why should more severe damage to semantic representations (indexed by verb comprehension scores) predict better chances of improvement? When reported, noun comprehension was below 84% in all individuals showing improved production of untreated verbs. One first consideration is that perhaps improvement was easier to measure in these individuals—they were likely to present with more severe impairment, and therefore could show more substantial changes. The fact they are more likely to improve for untreated items sheds light on the mechanisms of change. Semantic representations are thought of as sets of features (e.g., a pen is elongated, used to write, has ink inside, etc.), which are shared by several words (pencils are also elongated and used to write, but do not have ink inside). In word retrieval, these features activate several, meaning-related word forms (“used to write” will activate both/pen/and /pencil/). The lexeme with the largest semantic overlap is selected eventually (Patterson and Shewell, 1987; Bastiaanse and Van Zonneveld, 2004). In the event of partial loss of semantic knowledge, naming errors may occur due to insufficient activation of features that are critical to distinguish between related words, i.e., “has ink inside,” in our example (Caramazza and Hillis, 1990).

Due to the properties of semantic features just discussed, if this knowledge is disrupted by brain damage, and then restored by treatment, it can become available for the retrieval of all the words that share the same features. This will decrease error rates to both treated words and untreated words with shared features. Semantic Feature Analysis (SFA; Boyle and Coelho, 1995) was designed based on this rationale. In our dataset, the treatment techniques yielding improved production of untreated verbs in individuals with grammatical impairments who did not receive morphological cues include discussion of verb's semantic features (Rose and Sussmilch, 2008; Carragher et al., 2013), simultaneous semantic and gestural treatment, gesture-only treatment, repetition treatment (Rose and Sussmilch, 2008), and modified Constrained Induced Language Therapy (Maul et al., 2014). While not all of these treatment techniques required explicit discussion of semantic features, the occurrence of semantic priming provides evidence that semantic features are activated even if they are not explicitly discussed.

Altogether, the effects of morphological cueing, semantic impairment (indexed by noun comprehension) and grammatical impairment in determining generalization after verb therapies point to the specificity of treatment outcomes in relation to the levels of language processing engaged by the treatment task (Jacquemot et al., 2012), and the levels of language impairment (Miceli et al., 1996).

### The effect of session frequency on improvement for treated and untreated verbs

The finding that patients who received fewer therapy sessions per week were more likely to improve on both treated and untreated verbs is unexpected and contrasts with previous reports (e.g., Bhogal et al., 2003). At this stage, any attempt at an explanation is speculative, especially in the absence of many relevant details for each study (e.g., whether treatment was customized to each patient's needs or based on a “standard” protocol). For treated verbs, this apparently paradoxical effect of session frequency is observed only in individuals with relatively high scores on verb comprehension and word repetition tests. In the light of the discussion in Section Improvement of Lexical Retrieval of Treated Verbs, this could mean that patients with mild semantic damage, and in whom phonological processes, short-term memory and articulatory planning are relatively spared, have a greater potential for recovery and do not need frequent sessions (perhaps because they can learn the strategies applied during treatment sessions and apply them to more ecological circumstances of everyday life). However, it is not clear why the same (or a better) result cannot be obtained by increasing session frequency.

Similarly, we have no reasonable account for why patients with grammatical impairments who did not receive morphological cues and had severe noun comprehension problems were less likely to improve in the production of untreated verbs if they received more than 2.6 sessions per week. We note that, on average, patients who showed improvement for untreated verbs received a similar number of therapy sessions (12.96 and 12.45, respectively) but were in treatment for more days than those who did not show generalization (39.38 and 33.82, respectively). Since the number of treatment days also featured as a potentially informative predictor in the importance ratings, the seemingly paradoxical reverse frequency effect might reflect the overall duration of treatment. Indirect support for this interpretation comes from Dickey and Yoo (2010) who report that item-specific improvement occurs earlier in the course of treatment with a rapid and linear improvement, and generalization tends to appear later, and to show a slowly accelerating learning curve. It is then possible that these patients did not have enough therapy time. The relation of frequency, intensity, duration, and amount of sessions with response to treatment (for treated and untreated verbs) should be systematically examined in future studies.

Data on the “reverse” effect of session frequency are clearly counterintuitive and difficult to interpret. However, regardless of the mechanisms underlying it, the finding that improvement in the production of treated and untreated verbs is more likely in individuals who receive fewer therapy sessions challenges the general idea that more intensive treatment is more efficacious *per-se* (Bhogal et al., 2003). Reasons can be complex. For example, Brady et al. (2012) reported that patients tend to withdraw more often from intensive than non-intensive therapy. Further research should examine the complex nature of the relation between the frequency of weekly therapy and other treatment- and patient-related variables.

### Future directions

We provide tentative accounts for each of the identified predictors, in terms of cognitive mechanisms that potentially support improvement. Our interpretations are limited by theoretical and pragmatic issues. For example, even though we mentioned the potential role of cognitive, non-linguistic functions (e.g., STM) in determining treatment outcome, few studies reported results of cognitive screenings. Collecting this information is critical for future meta-analyses. A similar consideration applies to a number of known predictors of aphasia recovery. For example, information on socio-economic status, intelligence, pre-stroke ability to perform activities of daily living, stroke severity, lesion site and size, visuo-motor and attention skills, and sensitivity to priming was unspecified (or too vague to be used) in most studies. It is to be hoped that the diffusion of structural and functional neuroimaging techniques will help characterize brain structures and dynamics that contribute to specific types of recovery, and that an increasing number of potentially relevant variables will be taken into account.

It is also relevant to acknowledge a major challenge inherent in combining data from many different studies. Assessment scores as well as outcome measures were obtained from a variety of tests, and even when tests were similar in nature they may have differed in degree of difficulty. This may have introduced noise in our dataset. Unfortunately, many studies report on results of *ad-hoc* tests, but fail to provide normative values for them. In future treatment studies, it is imperative that normative data associated with these *ad-hoc* tests is also reported.

A separate issue relates to the reference literature used to interpret some of our findings. With respect to improved production of treated verbs, a substantial part of the literature used to support our interpretations comes from research with non-aphasic participants. The role of specific working memory processes in aphasia recovery should be independently established, in studies similar to Papagno et al. (1991)^4^. The same applies to episodic memory skills, that can help build up the effects of treatment. Auditory verb/noun comprehension may be disrupted by damage to distinct levels of processing, each of which may contribute to its predictive value, and should be examined in future studies. Finally, the relation between degree of preservation of semantic knowledge, sensitivity to priming, and potential for item-specific recovery must be confirmed empirically.

Similar considerations apply to improved production of untreated verbs. A finer-grained study of the effects of structural and morphological priming in aphasic individuals is a prerequisite for clearer interpretations. This issue may be examined in patients with different types of language impairment, in order to disentangle the roles of conceptual and grammatical features. Furthermore, the patients included in this meta-analysis suffer from heterogeneous and often underspecified grammatical difficulties. Our data shows that generalization is partially accounted for by the characteristics of language impairment presented by the patient. The heterogeneity and/or under-specification of the levels of language impairment in published reports may account for some unexpected results (e.g., Rose and Sussmilch, 2008 vs. Wambaugh et al., 2014), and should be explored in further research.

In addition, future studies may seek to identify predictors of other outcomes. Improved communication in daily living is the final goal of aphasia therapy, and has been shown to improve after treatment of verb production in sentences (e.g., Links et al., 2010). Further meta-analyses may help identify the predictors of transfer of treatment benefits to communication in daily life. Finally, we are not able to account for the inverse relation between frequency of treatment and language improvement. Considering the high impact that this variable has in provision of healthcare, it is crucial to assess its role in relation to other patient-related and treatment-related variables.

## Conclusion

Improved lexical retrieval of treated and untreated verbs occurs through different mechanisms. Improvement in production of treated verbs is observed in 76.1% of the patients. It may depend on restoring access to and/or rebuilding specific knowledge of lexemes. Success is determined by the availability of at least partial access to these representations (dependent on the activation of semantic features, indexed by verb comprehension scores), and by at least some ability to practice the labels to be restored or re-accessed (with short-term memory skills indexed by word repetition scores). The results on improved production of untreated verbs are less clear. This outcome is infrequent (14.5%), and likely signifies that in these cases treatment-related changes occurred at the level of processing abstract features (e.g., semantic and syntactic features), shared by different verbs. These features can be trained during therapy by using few lexical items and contexts. If recovered, they become available for a larger number of items, thereby facilitating encoding of grammatical information and, indirectly, access to untreated lexemes. Treatment techniques that engage processing of these features are associated with greater chances of generalization.

The present report should not be taken as an exhaustive list of all the factors and mechanisms that may be at play in recovery of treated and untreated materials. Our description of prognostic factors and their interactions is inevitably incomplete and preliminary. However, it already challenges a simplistic interpretation of some well-established predictors of recovery (e.g., session frequency, intensity, and aphasia severity). It invites to consider in detail the role of linguistic and language-related processes in determining effects of treatment. Our meta-analysis sheds some light on the mechanisms involved in different types of recovery, and can be used to inform theories and practices of therapy.

## Author contributions

VdA and GM contributed to the conception and design of the work. VdA, RB, and GB contributed to the definition of the data analysis procedures and to the interpretation of the data. VdA extracted and analyzed the data, wrote the first draft of the manuscript, and collated the input from all co-authors in subsequent versions of the manuscript. RB and GM added content to first draft and contributed with content and revisions to all sections of each subsequent version of the article. All authors approve the submitted version of the manuscript and agree to be accountable for all aspects of the work.

### Conflict of interest statement

The authors declare that the research was conducted in the absence of any commercial or financial relationships that could be construed as a potential conflict of interest.

## Appendix

### Example R code

In this study we ran all statistical analyses using R (R Core Team, 2015). The procedures are largely based on Tagliamonte and Baayen (2012). We used functions from the R packages randomForest, party, and lattice. These packages can be loaded with:

> library(randomForest)
> library(party)
> library(lattice)

Missing data was estimated using random imputation with:

>dat = rfImpute(database ~., data = metaAnalysis, iter = 100, ntree = 2000)

A random forest with unbiased conditional inference trees is obtained using:

> fit = cforest(OutcomeVar ~., data = dat, control = cforest_unbiased(mtry = 5, ntree = 5000))

We assessed the relative importance of predictors using conditional permutation variable importance:

> imp = varimp(fit, conditional = TRUE)

Plots with variables ordered by variable importance can be obtained with:

> dotplot(sort(imp))

For assessment of classification accuracy, the index of concordance C can be calculated using:

> fit.trp = treeresponse(fit)
> dat$PredFit = sapply(fit.trp, FUN = function(v)return(v[1]))
> dat$datFit = (dat$ImprUntreated = = “0”) + 0
> somers2(dat$PredFit, dat$datFit)

Conditional inference trees were produced with:

> MyTree = ctree(Outcome ~., data = dat);plot(MyTree)

^1^When discussing the effects of a treatment protocol, the term generalization may refer to improved lexical retrieval of untreated items (e.g., the training of *walking* enhances the retrieval of *running*) or to the increased use of treated morphological and syntactic processes, in contexts different to those presented with treated items. For example, training the production of regular past tense morphology by using the verb *to walk* results in improved production of regular past tense morphology of other, untrained regular verbs. Generalization may also refer to improved retrieval of treated items in an untreated task (e.g., improved retrieval of *walking* in a sentence production task after treatment of *walking* in an action naming task). Throughout the present manuscript, we use generalization to denote improved lexical retrieval of untreated items. ^2^Fink et al. (1992), Marshall et al. (1997), Weinrich et al. (1997), Marshall et al. (1998), Weinrich et al. (1999), Raymer and Ellsworth (2002), Wambaugh et al. (2002), Wambaugh et al. (2004), Webster et al. (2005), Edwards and Tucker (2006), Raymer and Kohen (2006), Raymer et al. (2006), Rodriguez et al. (2006), Kim et al. (2007), Raymer et al. (2007), Wambaugh and Ferguson (2007), Rose and Sussmilch (2008), Conroy et al. (2009a,b,c), Webster and Gordon (2009), Links et al. (2010), Boo and Rose (2011), Faroqi-Shah and Graham (2011), McCann and Doleman (2011), Harris et al. (2012), Carragher et al. (2013), Park et al. (2013), Maul et al. (2014), Wambaugh et al. (2014). ^3^Assuming that all other processes that precede the lexeme level occur successfully. ^4^Though PV (Baddeley et al., 1988) had been previously diagnosed with aphasia (Basso et al., 1982), by the time she was studied there were no identifiable language deficits.
